# Artemisinin pre-treatment fore cisplatin dosage enhances high grade urothelial carcinoma treatment in male albino mice via reverse gene expression modulation of FGFR3, HRAS, P53 and KDM6A

**DOI:** 10.1186/s12885-024-12683-y

**Published:** 2024-08-08

**Authors:** Silvia Botrous, Ayaat Elmaghraby, Samar El Achy, Yehia Mustafa, Salah Abdel-Rahman

**Affiliations:** 1 Department of Genetics, Faculty of Agriculture, Alexandria University, Alexandria, Egypt; 2 Department of Nucleic Acid Research, Genetic Engineering and Biotechnology Research Institute, City of Scientific Research and Technological Applications, Alexandria, Egypt; 3 Department of Surgical Pathology, Faculty of Medicine, Alexandria University, Alexandria, Egypt

**Keywords:** Artemisinin, Cisplatin, Urinary bladder cancer, High grade urothelial carcinoma, BBN, FGFR3, HRAS, P53, KDM6A

## Abstract

**Background:**

Urinary bladder cancer, is the 10th most common global cancer, diagnosed in over 600,000 people causing 200,000 deaths annually. Artemisinin and its derivatives are safe compounds that have recently been proven to possess potent anti-tumor effects in vivo, through inhibition of cancer cell growth. The aim of this study is to assess the efficiency of artemisinin as a cancer treatment alone and as a pre-treatment fore cisplatin therapy for high grade urothelial carcinoma.

**Methods:**

Sixty male albino mice were divided into six groups, and BBN was used to induce urinary bladder cancer. Blood samples were tested for renal functions and complete blood counts, kidney and urinary bladder tissues were harvested for histopathological examination. Total RNAs from urinary bladder tissues was collected, and gene expression of FGFR3, HRAS, P53, and KDM6A was quantified using qRT-PCR.

**Results:**

Compared to the induced cancer group, the results revealed that FGFR3 expression levels were down-regulated in the induced cancer group treated by artemisinin only and the induced cancer group pre-treated with artemisinin prior to cisplatin by ~ 0.86-fold and 0.4-folds, respectively, aligning with HRAS down-regulation by ~ 9.54-fold and 9.05-fold, respectively. Whereas, P53 expression levels were up-regulated by ~ 0.68-fold and 0.84-fold, respectively, in parallel with KDM6A expression, which is up-regulated by ~ 0.95-folds and 5.27-folds, respectively. Also, serum creatinine and urea levels decreased significantly in the induced cancer group treated by artemisinin alone and the induced cancer group pre-treated with artemisinin prior to cisplatin, whereas the induced cancer group treated by cisplatin their levels increased significantly. Moreover, Hb, PLT, RBC, and WBC counts improved in both cancer groups treated by artemisinin alone and pre-treated with artemisinin prior to cisplatin. Histologically, in kidney tissues, artemisinin pre-treatment significantly reduced renal injury caused by cisplatin. While Artemisinin treatment for cancer in bladder tissues reverted invasive urothelial carcinoma to moderate urothelial dysplasia.

**Conclusions:**

This study indicates that artemisinin demonstrated a significant effect in reversal of the multi-step carcinogenesis process of high grade urothelial carcinoma and could enhance the effect of cisplatin therapy using artemisinin pre-treatment.

## Background

Bladder cancer is the 10th most common cancer in the world, with an estimated 573,278 new cases per year, representing 3% of all cancers. Regarding cancer mortality, it ranks as the 13th cause of cancer-related mortality [1]. The commonest type of bladder cancer is urothelial carcinoma, accounting for more than 90% of bladder tumors [2]. The tumor stage significantly influences bladder cancer management protocols, affecting patient outcomes. Current chemotherapeutic protocols for advanced bladder cancer patients still lack a complete cure, necessitating the search for new therapies. A recent consensus molecular classification of bladder cancer has identified six clusters with varying prognostic outcomes [3]. A number of molecular alterations including mutations in FGFR3, RB1, HRAS, p53, TSC1 genes have been implicated in the pathogenesis of urothelial carcinoma and play an important role in determining the prognosis of these tumors as well as their response to therapy [4]. Superficial and muscle-invasive bladder urothelial carcinomas are two distinct entities according to the most recent molecular classification, since they exhibit distinct clinical, morphological and molecular phenotypes. Superficial urothelial carcinoma develops originally from hyperplastic urothelium, followed by urothelial dysplasia turning into low grade non-invasive carcinoma. However, invasive urothelial carcinoma progress from carcinoma in situ, through high grade non-invasive urothelial carcinoma, then invades the lamina propria, followed by the muscularis propria. The pathogenetic pathway of superficial noninvasive urothelial carcinoma is characterized by FGFR3, tyrosine receptor, HRAS and PI3KCA mutations. On the other hand, high grade muscle invasive urothelial carcinoma mainly involves alterations in tumor suppressor genes including: p53, Rb and p16 [5].

FGFR3 is an oncogene located on chromosome 4 encoding tyrosine kinase that plays an important role in cell growth, differentiation and survival. Moreover, FGFR3 gene mutations have been detected in 18% of urothelial carcinomas, 14% of uterine carcinosarcomas, 5% of esophageal cancers, 5% of ovarian cancers and 4% of endometrial cancers. FGFR3 gene transcript (circRNA product) is over expressed in bladder cancer and associated with increased cancer cell proliferation and migration [6]. HRAS gene belongs to the RAS family of oncogenes (H-Ras, N-Ras and K-Ras) which encodes for highly related monomeric membrane localized guanosine diphosphate/guanosine triphosphate binding (G) protein products namely H-Ras, N-Ras and K-Ras. Overactive HRAS proteins have been shown to direct cells for uncontrolled division, and bladder carcinogenesis. HRAS overexpression is being detected in recurrent non-invasive urothelial carcinoma [7].

P53 is a tumor suppressor gene which plays a key role in suppression of neoplastic transformation through cell cycle arrest, or apoptosis. Alterations of the p53 gene are common in bladder cancer 50%, and are more frequently detected in high grade invasive urothelial carcinomas. Additionally, loss of p53 function has been associated with progression to invasive disease and decreased overall survival [8, 9]. KDM6A gene is also a tumor suppressor gene and a member of the histone H3 lysine 27 demethylase gene family. KDM6A gene plays a central role in regulating the enhancer activity as part of the COMPASS-like complex and its function is critical for embryonic stem cell differentiation and tissue development. The highest frequency of KDM6A mutations was found in 26% of all urothelial carcinomas, 52% of which were superficial and 38% were muscle-invasive urothelial carcinomas.

BBN, a derivative of N-nitroso-di-N-butylamine found in tobacco smoke, is highly effective in inducing bladder cancer in mice due to its ability to cause DNA alkylation damage, leading to the accumulation of mutations [10]. Cisplatin chemotherapy is a metal-based anti-cancer drug that treats various solid malignancies by inhibiting DNA synthesis [11]. Cisplatin is commonly used in adjuvant therapeutic protocols for bladder cancer effectively reducing the rate of recurrence, and inhibit tumor progression in cases with bladder preservation protocols. The therapeutic regimen can increase the 5-year survival rate of patients with MIBC by up to 58–68%. Nevertheless, the overall response rate of cisplatin is only approximately 35% [12]. One of the major drawbacks for the use of cisplatin therapy is its high nephrotoxicity, which leads to dose limitation in cancer patients [13].

Artemisinin is an active ingredient obtained from Chinese medicine (Sweet wormwood, *Artemisia annua* L.). It was previously used for the treatment of malaria and was also recently found to have an antitumor effect. Artemisinin has proved to inhibit metastasis, invasion and angiogenesis, whilst inducing apoptosis and cell cycle arrest [14]. Several previous studies have confirmed that the artemisinin and its derivatives have an inhibitory effect on the viability of leukemia, colon, melanoma, breast, ovarian, prostate, central nervous system and renal cancer cells [15].

The objective of this study is to examine the efficiency of artemisinin as a cancer treatment alone and as a pre-treatment to cisplatin chemotherapy on high grade urothelial carcinoma in male albino mice by assessing biochemical parameters (kidney functions), hematological parameters (CBC) and histopathological examination (kidney and urinary bladder tissues), in addition to the expression of oncogenes (FGFR3 and HRAS) and tumor suppressor genes (P53 and KDM6A).

## Materials and methods

### Experimental animal model

Sixty male albino mice were randomly selected from animal house at Faculty of Pharmacy, Pharos University, Alexandria, Egypt. The 60 mice (Mus musculus), with weight from 25 to 30 gm, were transferred and housed at Medical Research Institute, Alexandria University, Egypt. The animals were kept in plastic cages (five animals/cage) in a room maintained at proper environmental conditions (temperature 25°C, humidity 50% and 12h light–dark cycle), then the animals were given free access food and water. The animals were observed daily for abnormal signs, and were acclimatized for two weeks before starting the experiment. The entire cohort was divided into six groups (A, B, C, D, E and F), each comprised 10 mice and the whole duration of the experiment spanned 15 weeks (Table 1). Group A was the control group, group B was the BBN induced group (0.05% of BBN dissolved in drinking water) [10], group C received artemisinin only (orally administered 200 mg/kg for 28 days) [16], group D was induced by BBN then received artemisinin (after induction), group E was induced by BBN then injected using cisplatin (intravenous injection administrated 1 mg/kg twice every week at intervals of 3, or 4 days for 2 weeks) [17], and group F was induced by BBN then received artemisinin (for 28days) followed by cisplatin injections 1mg /2times/week. It should be noted that the artemisinin was obtained from double wood supplements (Philadelphia, USA).

**Table 1:** Experimental animal design groups, number of animals and treatments

Group	Number of animals	Treatment (dose dissolved in 10 ml distilled water/30 g body weight/day)
Group A	10	Control (water + food)
Group B	10	0.05% BBN in drinking water
Group C	10	200 mg artemisinin
Group D	10	0.05% BBN + 200 mg artemisinin
Group E	10	0.05% BBN + 1 mg cisplatin
Group F	10	0.05% BBN + 200 mg artemisinin + 1 mg cisplatin

### BBN-induced mouse model of urinary bladder cancer

BBN (N-butyl-N-(4-hydroxybutyl)-nitrosamine) was used to induce urinary bladder cancer in male albino mice by giving tap water containing 0.05% BBN (Sigma–Aldrich, Dorset, UK) for 8 weeks maximum and then the BBN was replaced with tap water for four weeks until the end of 12th week. To detect the induced tumors by BBN, mice were sacrificed at 0, 1, 2, 4, 8 and 12th weeks [10]. Before sacrificing, the mice were anesthetized with intraperitoneal injection of 150–200 mg/kg amobarbital sodium by cervical dislocation. The urinary bladders were harvested and cut sagittal into two parts, one part was designated for RNA isolation and stored in liquid nitrogen immediately, the other part was stored in 4% paraformaldehyde for paraffin embedding and histological examination.

### Biochemical and hematological analysis

The analysis of blood samples was tested for kidney functions (urea and creatinine) and CBC. Where, the blood samples for the kidney functions were collected in serum coated tubes (Cobas-C & Cobas- E (Roche) 26-instrument, USA). While, in EDTA coated tubes (Swelab Alpha Basic, Sweden), blood samples were collected for CBC to assess four parameters: Hb, PLT, RBC and WBC. It should be noted that, the blood was centrifuged at 2000xg for 10 min to separate plasma.

### Histopathological studies

Kidney and urinary bladder tissues from all animal groups (A, B, C, D, E and F) were grossed and immediately fixed in 10% neutral buffered formalin for 24 h, then dehydrated in ascending grades of alcohol (70%, 80%, 95% and absolute alcohol), cleared by immersion in xylene and then impregnated in melted paraffin wax at 60°C for 1 h. The specimens were embedded in paraffin blocks, then 5 µm thick sections were cut and mounted onto clean glass slides. Sections were stained with the standard H&E stain, and examined using a light microscope.

### Real-time quantitative PCR (qRT-PCR)

Total RNAs from urinary bladder tissues of male albino mice, were isolated using Gene JET RNA Purification Kit (Geneaid Biotech, Korea), then quantified and qualified (purity) by using Nanodrop Spectrophotometer. Total RNAs samples were normalized (same concentration) to avoid any false increase in gene expression levels. Using SYBER Green 1-step qRT-PCR Kit (enzymomics, Korea), gene expression of oncogenes (FGFR3 and HRAS), tumor suppressor genes (P53 and KDM6A) (target genes) and GADPH (reference gene) were quantified by Real-Time PCR System (BioRad, USA) with the use of specific primers sequences (Forward/Reverse) and annealing temperature for each, as shown in Table 2. The qRT-PCR was performed in a reaction mixture of 10μl using 0.5μl TOPrealTM One-step RT qPCR enzyme mix, 5μl TOPrealTM One-step RT qPCR reaction mix (2X), 0.5μl forward and reverse primers (10pm), 0- 3.5μl water (PCR grade) and 50ng RNA template. qRT-PCR program was applied as one cycle of cDNA synthesis at 50°C for 30 min, one cycle of enzyme mix activation at 95°C for 10min and followed by 45 cycles of denaturation at 95°C for 5s., annealing at 55–60°C for 30s and extension at 72°C for 30s. The specificity of real-time PCR amplification was validated by analysis of melting curves, which ensured that only one PCR product was specifically amplified at the target size. The Ct was calculated for each replicate and final values were obtained from the average of three replicates per sample. The expression values of the studied genes were normalized using the expression values of a reference gene GADPH. Studied gene expression analysis was done using 2^−∆∆Ct^ method [22] Studied gene expression analysis was done using 2^−∆∆Ct^ method as follows:

ΔCt calibrator = Ct _GOI c_—Ct _norm c_

ΔCt sample = Ct _GOI s_—Ct _norm s_

ΔΔC = ΔCt _sample_—ΔCt _calibrator_

Fold difference = 2-ΔΔCt.

Where, calibrator: control, norm: housekeeping gene, GOI: gene of interest, S: sample and C: control. The significance differences between genes expression means were estimated by one-way ANOVA at significance level (*p* < 0.05).

**Table 2:** Primer sequences and annealing temperatures of FGFR3, HRAS, P53, KDM6A and GADPH genes

Gene	Primer sequence (forward/reverse)	Annealing temperature	Reference
FGFR3	5′-ACAGGTGGTCATGGCAGAAGCT-3′/ 5′-CTCCATCTCAGATACCAGGTCC-3′	60°C	[18]
HRAS	5′-TCGCACTGTTGAGTCTCGGCAG-3′/ 5′-TATGCTGCCGAATCTCACGGAC-3′	60°C	[19]
P53	5′-TGAAACGCCGACCTATCCTTA-3′/ 5′-GGCACAAACACGAACCTCAAA-3′	60°C	[20]
KDM6A	5′-AGCACAGAGGAGCCGTGGAAAA-3′/ 5′-GTCGTTCACCATTAGGACCTGC-3′	60°C	[21]
GADPH	5′-CCTCGTCCCGTAGACAAAATG-3′/ 5′-TGAAGGGGTCGTTGATGGC-3′	55°C	[20]

### Statistical analysis

Statistical analysis was performed using the statistical analysis software SPSS software (Version 22). Comparisons between groups were made using one-way ANOVA, followed by LSD test. Results are presented as means ± standard deviation (SD). Values of *p* < 0.05 were accepted as statistically significant and the corresponding graphs were generated by GraphPad Prism 10.

## Results

### Biochemical and hematological analysis

**Fig. 1:**
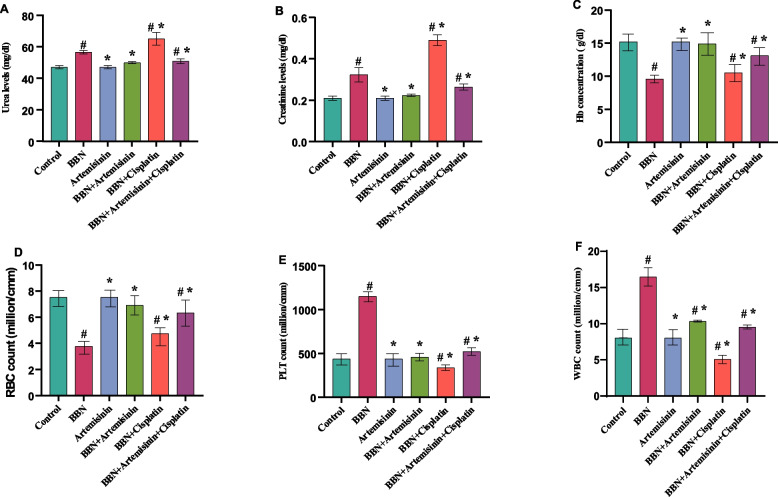
Biochemical and hematological analysis in studied groups. **A** urea levels, (**B**) creatinine levels, (**C**) Hb concentrations, (**D**) RBC count, (**E**) PLT count and (**F**) WBC count in. Data are expressed as mean ± SD where, #*P* < 0.05 vs Control, *P < 0.05 vs BBN

The results of the biochemical analysis revealed that urea and creatinine levels increased significantly in the B, E, and F groups compared to the control group (A). In contrast, the C, D, and F groups decreased significantly, whereas the E group increased significantly compared to the cancer group (B), as shown in Fig. 1A and B and Table 3. For hematological analysis of Hb concentration and RBC counts, the results showed a significant decrease in the B, E, and F groups compared to the control group (A). While, the C, D, E, and F groups increased significantly compared to the cancer group (B), as appears in Fig. 1C and D. PLT and WBC counts revealed a significant increase in the B and F groups, whereas the E group decreased significantly. Furthermore, the D group showed a significant increase only in WBC count as compared to the control group (A). In contrast, the C, D, E, and F groups decreased significantly compared to the cancer group (B), as illustrated in Fig. 1E and F.

**Table 3:** Biochemical and hematological analysis in the experimental animal groups

Test	Groups	A	B	C	D	E	F
Biochemical analysis							
** Urea**	M ± SD	47.00 ± 1.000	56.50 ± 1.081^#^	47.00 ± 1.000^*^	49.90 ± 0.754^*^	65.00 ± 4.000^#*^	50.83 ± 1.474^#*^
** Creatinine**	M ± SD	0.210 ± 0.01	0.323 ± 0.035^#^	0.210 ± 0.010^*^	0.223 ± 0.057^*^	0.490 ± 0.026^#*^	0.263 ± 0.015^#*^
**Hematological analysis**							
** Hb**	M ± SD	15.20 ± 0.010	9.57 ± 0.580^#^	15.20 ± 0.010^*^	14.90 ± 0.010^*^	10.50 ± 0.010^#*^	13.10 ± 0.010^#*^
** PLT**	M ± SD	435.0 ± 1.000	1150.0 ± 1.000^#^	435.0 ± 5.000^*^	458.3 ± 42.359^*^	335.6 ± 33.471^#*^	520.3 ± 44.467^#*^
** RBCs**	M ± SD	7.52 ± 0.010	3.76 ± 0.010^#^	7.52 ± 0.020^*^	6.91 ± 0.748^*^	4.76 ± 0.010^#*^	6.32 ± 1.005^#*^
** WBCs**	M ± SD	8.05 ± 0.010	16.46 ± 1.406^#^	8.05 ± 0.043^*^	10.34 ± 0.126^#*^	5.06 ± 0.568^#*^	9.53 ± 0.285^#*^

Values were expressed as the mean ± SD of experiment. The significant differences were tested by one-way ANOVA

#*P* < 0.05 vs Control

*P < 0.05 vs BBN

### Histopathological examination

#### Histological alterations in urinary bladder tissues during BBN-induction

**Fig. 2:**
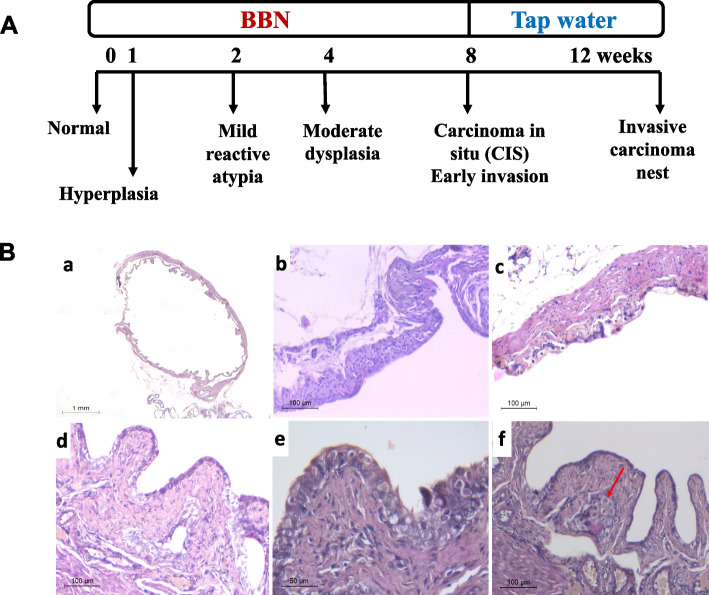
Histopathology of BBN-induced bladder cancer in mice. **A** A graphical illustration of the BBN treatment chronology. **B** Representative images demonstrating histological alterations. Where, (a) unremarkable urothelial lining with no changes, (b) hyperplastic urothelial lining with no atypia, (c) mild urothelial dysplasia, (d) moderate dysplasia with broad papillae lined by thickened urothelial lining, (e) full thickness dysplasia in the urothelial lining cells amounting to carcinoma in situ and (f) focus of invasive carcinoma nest in the lamina propria (red arrow)

To investigate the sequential urothelial changes in BBN-induced urothelial carcinoma, bladder tissue samples from male mice at various time points before (control group), during and after BBN treatment were examined (Fig. 2A). After one week of BBN (0.05%) administration, histopathological examination revealed a hyperplasic urothelial lining without atypia in the bladder specimens compared to the control group (Fig. 2B.a and b). After two weeks, mild urothelial dysplasia was detected (Fig. 2B.c). Moderate urothelial dysplasia as well as broad papillae lined by a dysplastic urothelium was observed after four weeks of BBN giving (Fig. 2B.d). Urothelial cells showed loss of polarity, pleomorphic, enlarged irregular and hyperchromatic nuclei. After eight weeks, the presence of full thickness dysplasia in the urothelial lining cells amounting to CIS was observed (Fig. 2B.e). At the end of the 12th-week, invasive urothelial carcinoma was detected, where invasive nests of neoplastic urothelial cells were identified within the lamina propria, as shown in Fig. 2B.f.

#### Post-treatment histological examination of the kidney and bladder tissues

**Fig. 3:**
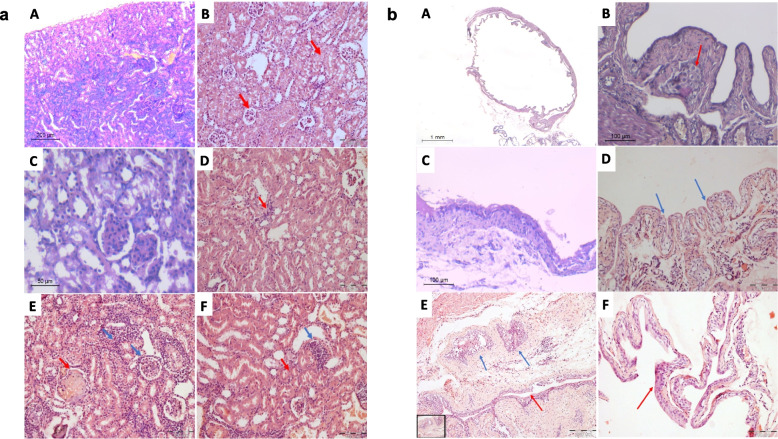
Histopathological examinations of kidney (**a**) and urinary bladder (**b**) tissues in studied groups. Where, (A) control group, (B) BBN (cancer group), (C) artemisinin group, (D) BBN + artemisinin group, (E) BBN + cisplatin group and (F) BBN + artemisinin + cisplatin group

After 8 weeks of using BBN induction of urinary bladder cancer, the treatment with artemisinin started (for 4 weeks) and subsequently with cisplatin (for 2 weeks) until the end of the experiment (at 15th week). Results of the histopathological examination of kidney tissues (groups A, B, C, D, E and F) revealed unremarkable pathological features, including patent glomeruli and normal renal tubules with absence of interstitial inflammatory infiltrates, or fibrosis as well as normal caliber blood vessels in the control group (Fig. 3a.A). In group B, interstitial nephritis was detected, with the presence of lymphoplasmacytic interstitial infiltrates. The renal tubules revealed cloudy swelling, as well as tubular casts. Glomerular mesangial hyper-cellularity was also noted as shown in Fig. 3a.B. In group C, the kidneys showed unremarkable pathological features with patent glomeruli and normal renal tubules, no vascular changes and no interstitial inflammation or tubular casts (Fig. 3a.C). In group D, the kidneys revealed mild interstitial inflammation and no vascular changes were seen, as shown in Fig. 3a.D. In group E, moderate to severe tubulointerstitial inflammation was evident. The glomeruli exhibited mesangial hypercellularity with focal segmental necrosis and the tubules revealed cloudy swelling, as well as tubular casts (Fig. 3a.E). In group F, tubulointerstitial nephritis was evident in the form of moderate to severe inflammatory cellular infiltrates with tubular injury, cloudy swelling, as well as tubular casts, as shown in Fig. 3a.F.

The results of the histopathological examination of the urinary bladder tissues showed unremarkable urothelial lining with no changes, no evidence of inflammatory cellular infiltrates in the submucosa and normal musculosa layer in control group (Fig. 3b.A). In group B, an invasive urothelial carcinoma was seen with an invasive nest within the lamina propria, as shown in Fig. 3b.B. In group C, there was an unremarkable urothelial lining and no evidence of inflammatory cellular infiltrates within the submucosa (Fig. 3b.C). In group D, moderate urothelial dysplasia (low grade) and a non-invasive papillary urothelial neoplasm was noted (blue arrows), as shown in Fig. 3b.D. Urothelial dysplasia and a focus of CIS, as well as invasive nests of malignant cells were seen in group E, as shown in Fig. 3b.E. Lastly in group F, focal dysplastic changes in the urothelium were found, as shown in Fig. 3b.F.

### Quantitative Real-time PCR gene expressions

#### FGFR3 and HRAS oncogenes

**Fig. 4:**
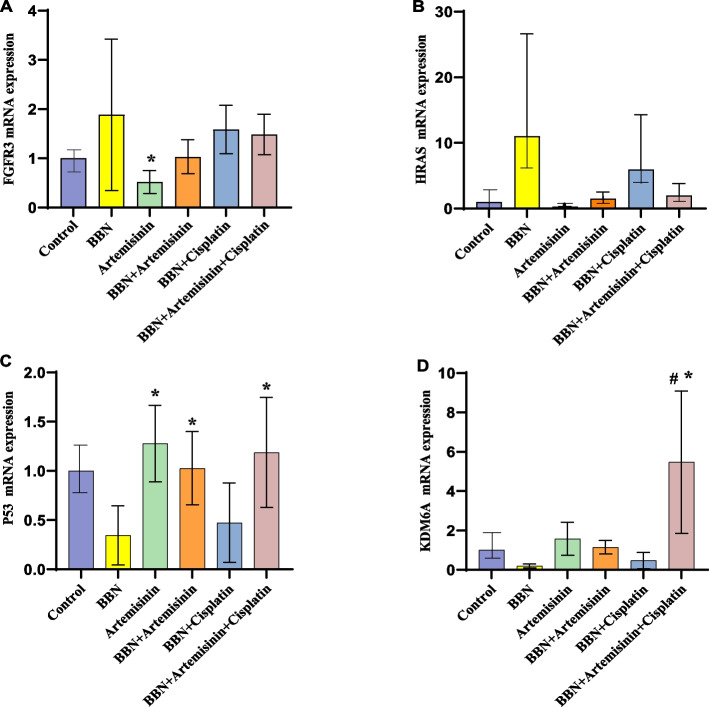
RT-qPCR was applied to detect the expressions of oncogenes (**A**) FGFR3, (**B**) HRAS and tumor suppressor genes (**C**) P53, (D) KDM6A in mouse urinary bladder tissues. Where, #*P* < 0.05 vs Control, *P < 0.05 vs BBN

In male albino mice's urinary bladder tissues, FGFR3 oncogene expression levels increased by about 0.88-fold, 0.02-fold, 0.58-fold, and 0.48-fold in the B, D, E, and F groups, respectively, whereas the C group decreased by ~ 0.48-fold compared to the control group (Fig. 4A). In contrast, the D, E, and F groups decreased by around 0.86-fold, 0.3-fold, and 0.4-fold, respectively, and the C group decreased significantly by ~ 1.36-fold compared to the cancer group (B). HRAS gene expression levels increased by ~ 10.02-fold, 0.48-fold, 4.94-fold, and 0.97-fold in the B, D, E, and F groups, respectively, whereas the C group decreased by ~ 0.72-fold compared to the control group (Fig. 4B). In contrast, the C, D, E, and F groups decreased by ~ 10.74-fold, 9.54-fold, 5.08-fold, and 9.05-fold, respectively, compared to the cancer group (B).

#### P53 and KDM6A tumor suppressor genes

The expression levels of the P53 tumor suppressor gene decreased by ~ 0.66-fold and 0.53-fold in the B and E groups, respectively, while the C, D, and F groups increased by ~ 0.27-fold, 0.02-fold, and 0.18-fold compared to the control group (Fig. 4C). In contrast, the C, D, and F groups increased significantly by around 0.93-fold, 0.68-fold, and 0.84-fold, respectively, and the E group increased by ~ 0.13-fold compared to the cancer group (B). KDM6A gene expression levels decreased by ~ 0.81-fold and 0.53-fold in the B and E groups, respectively, whereas the C and D groups increased by ~ 0.57-fold and 0.14-fold, respectively, and the F group significantly increased by ~ 4.46-fold compared to the control group (Fig. 4D). In contrast, the C, D, and E groups showed increases of around 1.38-fold, 0.95-fold, and 0.28-fold, respectively, while the F group increased significantly by ~ 5.27-fold compared to the cancer group (B).

## Discussion

The main objective of this study was to examine the activity of artemisinin as an anti-cancer treatment of the high-grade urothelial carcinoma whether administered alone or prior to chemotherapy (cisplatin). Where, the biochemical results revealed that urea and creatinine activities were improved in the BBN induced group treated with artemisinin alone and in the BBN, induced group pre-treated with artemisinin and then cisplatin compared to the induced group. These results revealed that using artemisinin has improved the side effects caused by BBN and cisplatin and reduced the urea and creatinine levels in high grade urothelial carcinoma-bearing mice. Similarly, a previous study reported that normal mice and breast cancer-bearing mice treated with artemisinin did not show any significant alterations in both creatinine and urea levels. Studies using cisplatin, reported a high nephrotoxic effect, leading to increasing of both creatinine and blood urea nitrogen levels [23]. Another study reported that artesunate (derivative of artemisinin) treatment, caused a significant reduction of the creatinine and blood urea nitrogen levels in cisplatin induced acute kidney injury in mice. A previous study found that the hematological results compared to the cancer induced group showed that Hb and RBC counts were improved in BBN induced mice treated by artemisinin alone and in the BBN induced group pre-treated with artemisinin, then cisplatin [24]. A previous study revealed that Cisplatin therapy showed slight improvement in BBN treated with artemisinin alone, with a decrease in PLT and WBCs. No significant changes in Hb, RBCs, and WBC counts in normal mice, but an increase in WBC counts in breast tumor-bearing mice [23]. However, another previous study showed that high doses of artesunate and gemcitabine treatment significantly affected hematological parameters in breast-cancer-bearing mice, reverting to normal values, but did not cause obvious injury or inflammation [25]. Another previous study reported that a single dose of cisplatin injection caused a significant decrease in the mean values of Hb, PLT, RBCs and WBCs compared to the control groups [26].

The results of the histopathological examination compared to the cancer group revealed that kidney tissues showed tubulointerstitial nephritis with acute tubular necrosis in the BBN group. While in the BBN group treated with cisplatin, moderate tubulointerstitial nephritis was observed. On the other hand, mild interstitial inflammation was observed in the BBN group treated with artemisinin. While mild reversible kidney injury and interstitial inflammation was observed in the BBN group pre-treated with artemisinin, then cisplatin. These findings demonstrate that artemisinin ameliorated tubulointerstitial nephritis and acute tubular necrosis in BBN-induced mice. Moreover, pre-treatment using artemisinin prior to cisplatin therapy significantly reduced renal damage caused by cisplatin in urinary bladder cancer-bearing mice. It worth mentioning that artemisinin is protective against renal cytotoxicity in rats subjected to cisplatin as it is already used with many chemotherapeutic protocols for various tumors in clinical practice. A previous study revealed that histopathological examination of the kidneys and testes showed no evidence of structural abnormalities in rats treated with artemisinin, in doxorubicin-induced renal and testicular toxicity [27]. Another previous study showed that artesunate (derivative of artemisinin) improved renal dysfunction in cisplatin-induced acute kidney injury, and the results demonstrated that the artesunate significantly decreased renal damage [24].

On the histological level, the current study deals with an invasive high-grade urothelial carcinoma model, which did not reach the muscle invasive stage, but would have, should it have been left for a longer time period. Invasive urothelial carcinoma invading the lamina propria was seen in the BBN group. While urothelial dysplasia and focal CIS were detected in the BBN group treated with cisplatin, On the other hand, moderate urothelial dysplasia and non-invasive papillary urothelial neoplasia was observed in the BBN group treated with artemisinin. Only focal dysplastic changes of the urothelium were found in the BBN group pre-treated with artemisinin followed by cisplatin. Our findings revealed that artemisinin exhibited a significant effect in reversing the carcinogenetic pathway of high grade urothelial carcinoma, and this efficiency was greater magnified by artemisinin pretreatment. in previous study explained that treatment with artesunate at doses of 20, 100, and 200 mg/kg, significantly reduced tumor sizes in a dose-dependent manner. This finding suggests that the artesunate may have a potential therapeutic effect on bladder cancer. In another in vitro study, the authors reported that artesunate significantly inhibited tumor cell growth and proliferation in a time- and dose-dependent manner in cisplatin-sensitive, and cisplatin-resistant bladder cancer cell lines [16, 28]. Another similar study declared that artesunate exhibited significant anti-tumor effect on lung cancer A549 cells and this efficiency was enhanced on combination with cisplatin [29]. According to [30], the combination of artesunate and cisplatin showed promising anti-tumor activity against head and neck cancer cell lines, even at low cisplatin doses.

Concerning gene expression results compared to the BBN induced cancer group, FGFR3 and HRAS (oncogenes) showed strong down-regulated expression levels in both BBN treated with artemisinin group and BBN + cisplatin group pre-treated by artemisinin. While, slight down-regulation of expression levels was observed in the BBN group treated with cisplatin alone. In contrast, P53 and KDM6A (tumor suppressor genes) showed significant up-regulation expression levels in BBN treated with artemisinin group and BBN + cisplatin group pre-treated by artemisinin. While, slightly up-regulating expression levels were observed in BBN group treated with cisplatin alone. A previous study, demonstrated that dihydroartemisinin (derivative of artemisinin) increased apoptosis in non-small cell lung cancer cells with FGFR, or RAS mutations [31]. Another study found that after cisplatin treatment intermediate and low levels of FGFR3 and FGFR4 were expressed in different head and neck squamous cell carcinoma cell lines [32].

In a previous study, KDM6A expression and its downstream effects were found to be lost in bladder cancer as a result of inactivating mutations. KDM6A-deficient cells are dependent on the function of another protein, called Enhancer of EZH2, which is often over-expressed and associated with poor prognosis in muscle-invasive bladder cancer. Therefore, EZH2 inhibition, delayed tumor onset in KDM6A-null cells and caused regression of KDM6A-null bladder tumors in multiple mouse models [33]. In another related study, the authors also reported a high sensitivity of bladder cancer cells to EZH2 inhibition alone, or in combination cisplatin treatment in the presence of KMD6A and SWI/SNF mutations. However, and according to the findings in our study, we suggest that EZH2 inhibition could be enhanced via artemisinin alone and affected by pretreatment before cisplatin dosage treatment [34]. For p53, in a previous study, *Artemisia annua* L. extract (source of artemisinin) enhanced the up-regulation of p53 and p63 expression, with a promising in vivo anticancer effect. Thus, the authors suggested that *Artemisia annua* could be used as a complementary therapy in breast cancer treatment protocols [35]. In another study indicated that artemisinin up-regulated p53 expression through the ERK1/2 signaling pathway. The authors also revealed that artemisinin exhibited direct anti-proliferative effect on gastric cancer cell lines, and suggested it might be a good candidate for clinical trials in gastric cancer treatment [36].

The recent molecular classification of urothelial carcinoma divides it into two main pathogenetic pathways: low-grade noninvasive urothelial carcinoma, harboring mainly FGFR3 mutations that consequently affect FGFR3 expression, and high-grade invasive urothelial carcinoma, characterized by mutations in tumor suppressor genes like p53 and KDM6A that consequently affect their expression [37]. In light of this classification, the current study model complies with the high-grade invasive subtype. However, FGFR3 and HRAS expression levels have been markedly down-regulated in high-grade invasive urothelial carcinoma in treated groups with artemisinin, in contrast to p53 and KDM6A expression levels, indicating artemisinin treatment reversed the multistep carcinogenesis process to an early pre-invasive stage that can be treated as superficial bladder tumors with space for targeted FGFR3 therapy. Both histomorphology and molecular features validate that this model is a high-grade invasive type of urothelial carcinoma, with current treatment protocols being adjuvant chemotherapy and/or immunotherapy. The research suggests an alternative therapy for this subset of tumors, not all bladder cancer.

## Conclusions

The study shows that artemisinin treatment and pre-treatment, followed by cisplatin, significantly improves biochemical and hematological markers in high-grade urothelial carcinoma-bearing mice. Artemisinin pre-treatment protects against cisplatin nephrotoxicity and reverses carcinogenesis in bladder cancer. It down-regulates FGFR3 and HRAS expression, while up-regulating P53 and KDM6A expression. The findings suggest that artemisinin can reverse the multi-step carcinogenesis process in high-grade urothelial carcinoma.

## Data Availability

The datasets used and/or analysed during the current study are available from the corresponding author on reasonable request.

## Abbreviations

BBN: N-butyl-N-(4-hydroxybutyl) nitrosamine
CBC: Complete Blood Count
CIS: Carcinoma in Situ
Ct: Cycle Threshold
DR5: Death Receptor 5
EZH2: Zeste Homolog 2
H&E: Hematoxylin and Eosin
Hb: Hemoglobin
HRAS: Harvey-Ras
KDM6A: Lysine-specific demethylase 6A
KMT2A: Lysine N-methyltransferase 2A
KRAS: Kristen-Ras
MIBC: Muscle-Invasive Bladder Cancer
N-Ras: Neuroblastoma-Ras
PLT: Platelets
qRT-PCR: Quantitative Real-time PCR
RBCs: Red Blood Cells
SPSS: Statistical Package for Social Sciences
SWI/SNF: Switch/Sucrose Non-Fermentable
WBCs: White Blood Cells
